# *In Situ* Gene Mapping of Two Genes Supports Independent Evolution of Sex Chromosomes in Cold-Adapted Antarctic Fish

**DOI:** 10.1155/2013/243938

**Published:** 2013-02-12

**Authors:** Laura Ghigliotti, C.-H. Christina Cheng, Céline Bonillo, Jean-Pierre Coutanceau, Eva Pisano

**Affiliations:** ^1^Department of Earth, Environment and Life Sciences (DISTAV), University of Genoa, 16132 Genoa, Italy; ^2^Department of Animal Biology, University of Illinois, Urbana-Champaign, IL 61801, USA; ^3^SSM and CNRS UMR 7138, Département Systématique et Evolution, MNHN, 43 rue Cuvier, 75231 Paris, France

## Abstract

Two genes, that is, 5S ribosomal sequences and antifreeze glycoprotein (AFGP) genes, were mapped onto chromosomes of eight Antarctic notothenioid fish possessing a X_1_X_1_X_2_X_2_/X_1_X_2_Y sex chromosome system, namely, *Chionodraco hamatus* and *Pagetopsis macropterus* (family Channichthyidae), *Trematomus hansoni*, *T. newnesi*, *T. nicolai*, *T. lepidorhinus*, and *Pagothenia borchgrevinki* (family Nototheniidae), and *Artedidraco skottsbergi* (family Artedidraconidae). Through fluorescence *in situ* hybridization (FISH), we uncovered distinct differences in the gene content of the Y chromosomes in the eight species, with *C. hamatus* and *P. macropterus* standing out among others in bearing 5S rDNA and AFGP sequences on their Y chromosomes, respectively. Both genes were absent from the Y chromosomes of any analyzed species. The distinct patterns of Y and non-Y chromosome association of the 5S rDNA and AFGP genes in species representing different Antarctic fish families support an independent origin of the sex heterochromosomes in notothenioids with interesting implications for the evolutionary/adaptational history of these fishes living in a cold-stable environment.

## 1. Introduction

In fishes the processes that influence the definition of sex (sex determination) may be subject to intrinsic genetic control, environmental control, or a combination of both [1, 2]. When only genetic factors influence the direction of sex determination, a genetic system of sex determination (GSD) is active. In this case, the chromosomes bearing the sex determining gene/s can be defined as “sex chromosomes.” These chromosomes derived from proto-sex chromosomes (autosomes that supposedly contain a cluster of closely linked sex genes) mainly through suppression of recombination between male and female regions leading to chromosomal sex inheritance pattern (e.g., [2–6]). During their evolutionary history, the sex chromosomes might become morphologically distinct (heteromorphic) as the result of the extension of the region where recombination is suppressed, along with chromosomal changes often accompanied by heterochromatin increase. The presence of heteromorphic sex-related chromosomes, recognizable in a given species, is strongly indicative of genetic control of sex determination acting in that species over other theoretical sex control systems [1, 7, 8].

In fishes sex chromosomes are not often recognizable based on gross morphology, and only a minority of species show visible sex-specific heteromorphic chromosomes [1, 10–13]. In contrast, a surprisingly high frequency of morphologically differentiated sex chromosomes occur in Notothenioidei, a perciform fish group endemic to Antarctic waters [14, 15].

Of the approximately 130 notothenioid species in eight families known to date, 123 species and five families (Harpagiferidae, Nototheniidae, Artedidraconidae, Bathydraconidae, and Channichthyidae) form an Antarctic clade [9] and dominate the ichthyofauna of High Antarctic regions in terms of species number and biomass [16]. The evolution of the Antarctic notothenioid fishes in geographical isolation and in a chronically cold marine environment resulted in stenothermality of the extant species, and in the acquisition of a suite of adaptive modifications (e.g., [17–19]). Indeed, the species of the Antarctic notothenioid clade are able to live and thrive in the freezing and icy Antarctic waters thanks to remarkable cold adaptations, one of the most striking being the capability to produce antifreeze glycoproteins (AFGPs) [20–22].

The main aim of the present study was to provide a first characterization of the sex chromosomes of Antarctic notothenioid fishes in terms of gene content. We report here on the mapping of two genes, that is, 5S ribosomal sequences and antifreeze glycoprotein (AFGP) genes, onto the chromosomes of eight Antarctic notothenioid species, namely, *Chionodraco hamatus* and *Pagetopsis macropterus *(family Channichthyidae)*, Trematomus hansoni*, *T. newnesi*, *T. nicolai*, *T. lepidorhinus*, and *Pagothenia borchgrevinki* (family Nototheniidae), and *Artedidraco skottsbergi* (family Artedidraconidae), showing multiple sex chromosome systems of the X_1_X_1_X_2_X_2_/X_1_X_2_Y type. We uncovered differences in the gene content of the Y chromosomes in the eight probed species, referable to three patterns of Y and non-Y chromosome association of the 5S rDNA and AFGP genes. Remarkably, 5S rDNA sequences were found into the Y chromosome in the species *C. hamatus* and AFGP gene clusters were located on the Y chromosome of *P. macropterus. *Our results are the first information on the gene content of sex chromosomes in these cold-adapted fishes. 

## 2. Materials and Methods

### 2.1. Animal Sampling and Chromosome Preparation

Specimens were collected in the Ross Sea and Adélie Land coastal areas during Italian, American, and French Antarctic expeditions, from 2000 to 2010. Sites and size of sampling are summarized in Table 1. 

Fishes were kept in aquaria with running, aerated seawater, and treated *in vivo *with colchicine. Mitotic somatic cells were obtained from head kidney and spleen, following standard protocols for direct chromosome preparations in Antarctic fishes, fixed in 3/1 methanol/acetic acid (v/v), and stored at −20°C for later analyses. 

The experiments followed ethical protocols and anesthesia was administered prior to sacrificing the animals.

Voucher specimens of every studied species are conserved at the National Natural History Museum (MNHN, Paris) and at the Antarctic National Museum (University of Genoa).

### 2.2. FISH Probes

The 5S ribosomal probe corresponds to a partial sequence (87 bp) of the highly conserved 5S rRNA coding region, obtained from the genomic DNA of *Chionodraco hamatus* as described by Ghigliotti et al. [23]. 

A DNA fragment (1218 bp), obtained from the notothenioid *Dissostichus mawsoni* shotgun library of the BAC plasmid DmBAC64 ([22], AFGP gene H2A7 in GenBank accession number HQ440760), and encoding for the repetitive (Thr-Ala/Pro-Ala)n AFGP polyprotein sequence, was used as the AntiFreeze GlycoProtein (AFGP) probe. 

The 5S rDNA and the AFGP probes were nick translation labeled with biotin-16-dUTP (Roche) according to standard procedures, ethanol purified, and dissolved in the hybridization buffer (50% formamide/2X SSC, 40 mM, KH2PO4, 10% dextran sulphate) to a final concentration of 15 ng/*μ*L and 20 ng/*μ*L respectively.

### 2.3. Fluorescence *In Situ* Hybridization

The chromosomal preparations, aged for two days at room temperature, were denatured by heating at 70°C for 1 min in 70% (v/v) formamide/2X SSC (pH 7), dehydrated in a cold ethanol series, and air dried. 

The probes were denatured by heating at 75°C for 10 min, applied to chromosomal spreads (20 *μ*L per slide), and incubated overnight in a moist chamber at 37°C. Posthybridization washes were performed at 43°C: twice in 50% (v/v) formamide/2X SSC, twice in 2X SSC, and once in 4X SSC-Tween-20, for 5 min each. Bound probe was detected by incubation with streptavidin-Cy3 (Amersham Biosciences). The chromosomes were counterstained in 0.3 *μ*g/mL DAPI/2X SSC and mounted in a standard antifade solution (Vector). 

### 2.4. Conventional Chromosome Banding

Conventional banding protocols were applied to the chromosomes of the two species differing from all the other probed species after FISH (the two Channichthyidae species, *C. hamatus* and *P. macropterus*) in order to gain structural details on the sex chromosomes. Chromosome spreads on microscope slides were DAPI (4,4′,6-diamidino-2-phenylindole) stained according to standard protocols. A characterization of the pattern of chromosome heterochromatin was performed through the C-banding method [28]. Chromosome morphology was determined according to the centromere position following the nomenclature by Levan et al. [29]. 

### 2.5. Image Processing

Metaphase spreads were examined with an Olympus BX61 microscope equipped with a SenSys CCD camera (Photometrics). Photomicrographs were processed by CytoVision Genus software (Applied Imaging) and by the use of Adobe Photoshop software.

## 3. Results

### 3.1. FISH Mapping of 5S rDNA and AFGPs Genes

**Figure 1:**
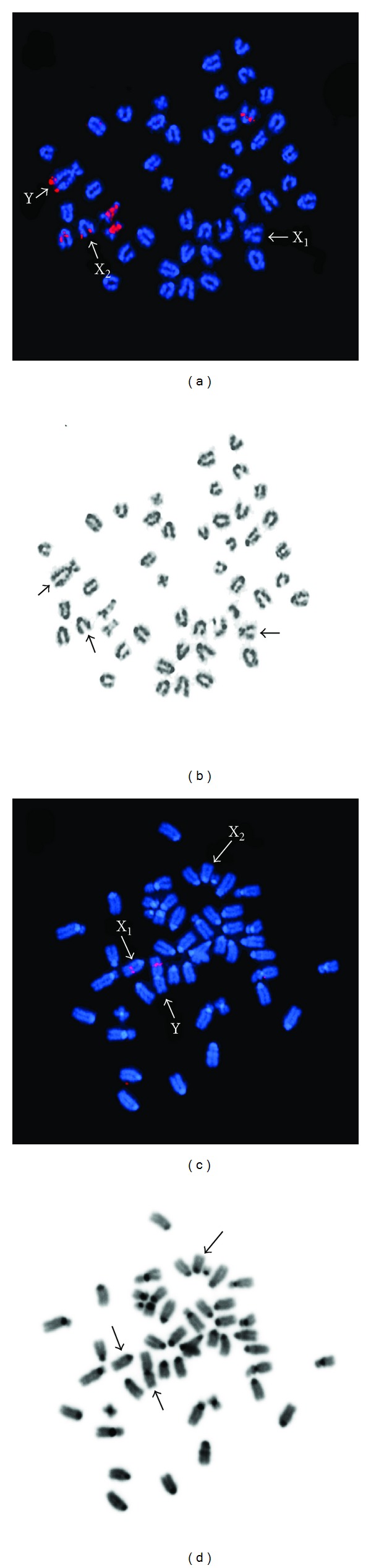
FISH analysis of male metaphase chromosomes in *C. hamatus* and *P. macropterus. C. hamatus*, metaphase plate after FISH with 5S rDNA probe (a) and reversed black and white DAPI staining (b); *P. macropterus* metaphase plate after FISH with an AFGP gene probe (c) and reversed black and white DAPI staining (d). Arrows indicate the chromosomes involved in the multiple sex chromosome system.

Fluorescence *in situ* hybridization with a 5S rDNA probe resulted in a consistent single signal on a pair of submetacentric chromosomes in all trematomid species and in multiple signals onto the chromosomes of the two channichthyidae species (results summarized in Table 2). In one of the icefish species, *C. hamatus*, the probe hybridized on the long arm of the Y chromosome, at peritelomeric position (Figures 1(a) and 1(b)). 

The AFGP probe hybridized at a single chromosomal position, on a pair of acrocentric chromosomes in the majority of the probed species. *P. macropterus* male differs from the others in showing hybridization signals at interstitial position on the Y chromosome and on a single acrocentric chromosome. Comparative analysis of the arms bearing the AFGP genes in *P. macropterus* showed that the signals on the Y chromosome and the acrocentric chromosome occupy a similar position with respect to the centromeres (Figures 1(c) and 1(d)). 

### 3.2. Conventional Banding Analysis of *C. hamatus* and *P. macropterus* Chromosomes

**Figure 2:**
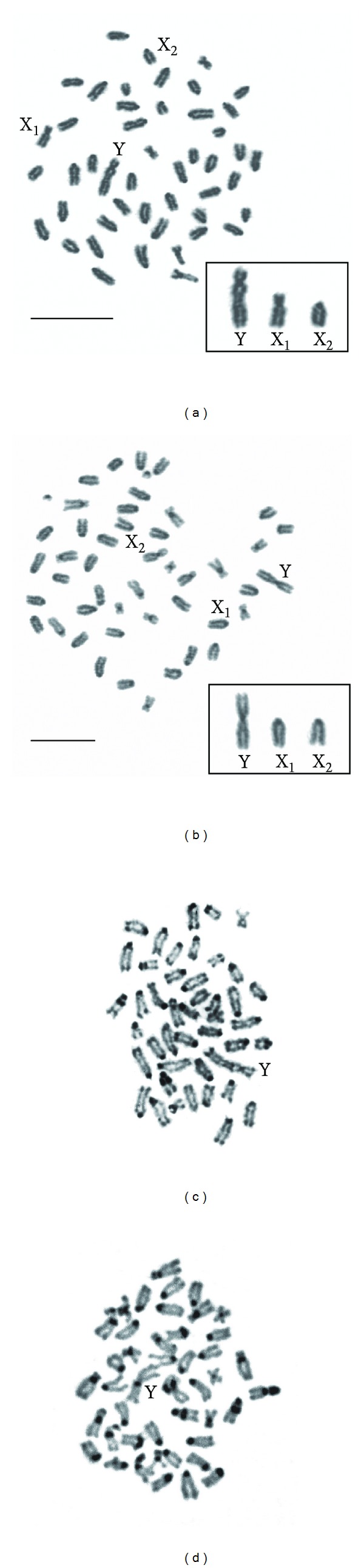
*C. hamatus* and *P. macropterus* reversed black and white DAPI- stained metaphases ((a) and (b), resp.) and C-banded chromosomes ((c) and (d), resp.). The sex chromosomes are labeled in the metaphase plates and shown enlarged separately in the boxes. The presumed X_1_ and X_2_ have been identified according to morphology, size, and banding pattern. Scale bars = 10 *μ*m.

Our analysis confirmed a diploid number 2*n* = 48 in the female specimens, and 2*n* = 47 in the males of both Channichthyidae species. The morphology of the male- and female-specific karyotypes in both the species has been previously reported [27]. The odd diploid number in the males was always coupled with the presence of a multiple X_1_X_2_Y sex system (Figure 2). 

*C. hamatus* male showed a sex linked heterochromosome system composed of a large submetacentric Y chromosome, a medium-sized submetacentric X_1_ chromosome, and an acrocentric X_2_ chromosome (shown enlarged in Figure 2(a)). X_1_ is unambiguously recognizable in the metaphase plates based on its morphology (submetacentric), size (medium sized), and banding pattern (homogeneously stained by DAPI); the X_2_ chromosome, similar to in morphology and size to several other acrocentrics in the karyotype, is identified by the subtelocentric position of 5S rDNA sequences after FISH mapping. The sex linked heterochromosome system of *P. macropterus* male consists of a large metacentric Y chromosome and two acrocentric chromosomes (X_1_ and X_2_) (shown enlarged in Figure 2(b)). In this species X_1_ and X_2_ were firstly assigned based on morphology (acrocentric) and size; *in situ* hybridization with an AFGP genes probe confirmed the occurrence of a large region of homology between the larger arm of Y chromosome and one acrocentric, thus allowing to assume the latter as the chromosome X_2_. 

The C-banding revealed heterochromatic blocks in most of the centromeres on the chromosomes of both species (Figures 2(c) and 2(d)). In *C. hamatus*, heterochromatic blocks were also detected on some of the telomeres, on the small arm of a pair of chromosomes, and at pericentromeric position on at least four chromosomes. A C-band is present on the long arm of the Y chromosome at interstitial position (Figure 2(c)). In *P. macropterus*, heterochromatic blocks were mainly detected at centromeric and pericentromeric position; large heterochromatic bands were also found on the entire short arms of a pair of submetacentric chromosomes. Only centromeric constitutive heterochromatin was detected on the Y chromosome of this species (Figure 2(d)). 

## 4. Discussion

In fishes, sex chromosomes are not often recognizable, unless detailed genetic and FISH-based cytogenetic studies are applied [30]. Among teleosteans, only a minority of species show heteromorphic sex-specific chromosomes [1, 10–13]. By contrast, the number of Antarctic notothenioid species possessing heteromorphic sex chromosomes is surprisingly high [14, 15]. 

In a longstanding cytogenetic screening effort of Notothenioidei, 45 notothenioid species living in the shelf waters of the High Antarctic Zoogeographic Zone and in the Southern part of the Seasonal Pack-ice Antarctic Zoogeographic Zone [31] have been cytogenetically studied. Out of those, 12 (26%) were found to have multiple sex chromosomes of the X_1_X_1_X_2_X_2_/X_1_X_2_Y type (Table 3).

The available data on the sex chromosomes in Antarctic notothenioid species has been previously described mainly through conventional cytogenetic techniques. At present, very little is known about their gene content [32–34], although this kind of structural cytogenomic information could allow to recognize the X_1_ and X_2_ chromosomes, thus providing clues for inferring the steps of the multiple sex chromosome system origin in the various species.

The herein described chromosomal location of 5S rDNA and AFGP genes on the Y-chromosomes of two channichthyidae species, *C. hamatus *and *P. macropterus*, respectively, contributes to improve the bulk of data on the gene content of sex chromosomes in Antarctic notothenioids and represents a starting point for reconstructing the chromosomal rearrangements that led to the formation of the secondary Y-chromosome in these two icefishes*.*

**Figure 3:**
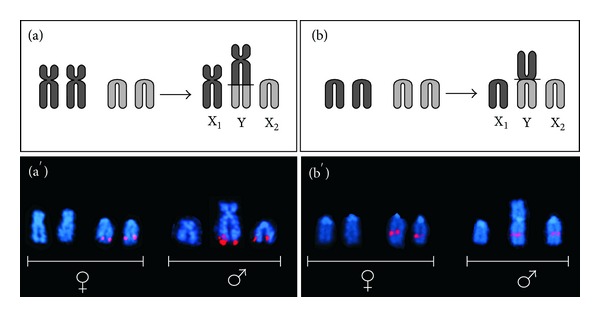
Schematic representation of the tandem fusion (a) and centric fusion (b) originating the Y-chromosome from a supposed ancestral condition in *C. hamatus* and *P. macropterus*, respectively. The black bars on the ideogrammatic Y chromosomes indicate the sites of tandem or centric fusion on the secondary-Y chromosomes. The sex chromosomes from female (conserving the supposed ancestral condition) and male *C. hamatus* (a′) and *P. macropterus* (b′) are shown after *in situ* hybridization with a 5S rDNA probe and AFGP genes probe, respectively.

Hypothetical mechanisms of origin of the secondary Y-chromosome in Channichthyidae species had been suggested by Morescalchi et al. based on morphological traits [27]. Although not providing the final proof, our findings are in agreement and provide support to previous hypotheses. The supposed mechanisms of origin of the sex chromosomes in the male of *C. hamatus *and *P. macropterus* are schematically illustrated in Figures 3(a) and 3(b), respectively. 

In *C. hamatus,* the peritelomeric position of 5S rDNA repeats on the long arm of the Y chromosome, and at the same position in an acrocentric, supports the origin of the secondary-Y by tandem fusion of a submetacentric and an acrocentric chromosomes (Figure 3(a)). This is also supported by the localization of 5S rDNA sequences at peri-telomeric position on a pair of acrocentric chromosomes in the female (Figure 3(a′)), supposedly conserving the ancestral condition preceding the evolution of the heteromorphic sex chromosomes. 

Similarly, the interstitial position of AFGP genes on an acrocentric chromosome and on the Y chromosome in *P. macropterus* male supports centric fusion between two acrocentrics of similar size as the most likely chromosomal rearrangement creating the secondary-Y in this species (schematized in Figure 3(b)). The chromosomal location of AFGP repeats in the same arm position on a pair of acrocentric chromosomes in the female (Figure 3(b′)) provides further support to this hypothesis. 

Chromosomal rearrangements, that is, tandem or centric fusions, seem therefore to have been crucial events in the formation of the sex linked heterochromosomes in the two icefishes. This is in accordance with a recent hypothesis that postulates a major role of chromosomal rearrangements themselves for the origin of multiple sex chromosome systems against a diminished or immaterial role of heterochromatinization [35]. Indeed, non significant amounts of constitutive heterochromatin have been detected through C-banding on the Y-chromosomes and/or on the X_1_ and X_2_ chromosomes of the two icefishes (Figures 2(c) and 2(d)). Notwithstanding, we cannot exclude that the presence of repetitive DNAs (5S rDNAs and AFGP sequences) onto the sex chromosomes of *C. hamatus* and *P. macropterus* would be a factor predisposing to further structural changes of the sex chromosomes. Indeed accumulation of repetitive sequences might occur, as the Y-chromosomes further evolve, as a consequence of the reduced rate of recombination between the sex chromosomes. 

This first data on the gene content of the sex chromosomes in the two icefish species are relevant for tracing the architectural evolutionary changes of their Y-chromosomes. Moreover, the chromosomal location of 5S rDNA and AFGP genes provided a more general remarkable information: three patterns of association of these genes to the Y-chromosomes are recognizable in the eight Antarctic notothenioid species (see results summarized in Table 2). The distinctive differences revealed by the FISH analysis support the hypothesis that sex linked heterochromosomes likely arose independently in notothenioid fishes. 

**Figure 4:**
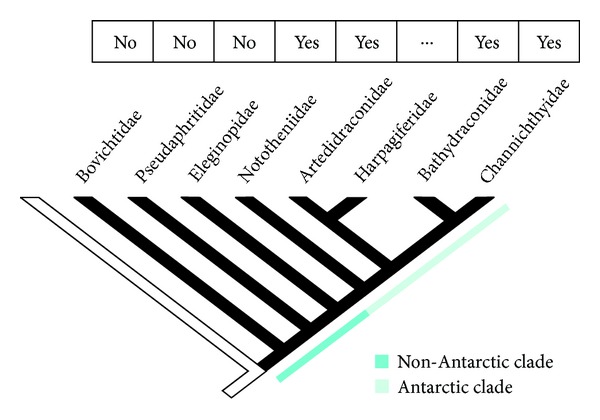
Occurrence of heteromorphic sex chromosomes in the notothenioid families (phylogeny according to [9]).

Interestingly, according to present information, heteromorphic sex chromosomes occur only in species of the Antarctic clade (families Artedidraconidae, Nototheniidae, Bathydraconidae, and Channichthyidae) (Figure 4), that experienced life in chronically cold marine waters over 10–14 million years. No morphologically differentiated sex chromosomes were ever found in species belonging to the non-Antarctic notothenioid families (Bovichtidae, Pseudaphritidae, and Eleginopidae) [36, 37], distributed in the sub-Antarctic region of the Southern Ocean, where they had not been exposed to natural selection by freezing marine conditions in their evolutionary history [17]. The occurrence of heteromorphic sex chromosomes exclusively in Antarctic members of the Notothenioidei suborder might have interesting evolutionary/adaptive implications.

Considering the presence of cytogenetically distinct sex chromosomes as an evidence of a genetic control of sex determination stable for some time in a species [1, 7], the occurrence of sex heterochromosomes in Antarctic notothenioid species (and not in their non-Antarctic relatives), the high frequency of sex heterochromosomes in the Antarctic fish, and the independent origination of sex chromosomes in stenothermal notothenioids are suggestive of a convergent evolutionary trend towards a genetic sex determination system in Antarctic notothenioids. Indeed, the prominence of genetic control of sex determination seems adequate to assure proper sex ratio in an invariably cold and geographically isolated environment such as coastal Antarctic regions where these fishes live. 

The current scanty information on the population genetics of notothenioid species prevents us from evaluating the influence of population genetic parameters that also might have played a role in the fixation of new chromosomal rearrangements leading to the formation of sex chromosomes. 

A wider documentation on the frequency of sex linked chromosomes in a comprehensive sampling of Antarctic notothenioid taxa, the increase of population genetic data, and the use of new genomic tools that are being made available for Notothenioid fishes [19, 38, 39] will be the next steps to be walked on the way to investigate this intriguing evolutionary/adaptive hypothesis. 

## Figures and Tables

**Table 1:** Information on the site of sampling and size of the samples used in the cytogenetic analyses. M: male, F: female.

Species	Sampling site	*N*
*Chionodraco hamatus *	Ross Sea Adélie Land	16M/17F 2M/1F
*Pagetopsis macropterus *	Ross Sea	4M/1F
*Trematomus hansoni *	Ross Sea	9M/11F
*T. newnesi *	Ross Sea	10M/15F
*T. nicolai *	Ross Sea	1M/1F
*T. lepidorhinus *	Ross Sea	3F/2M
*Pagothenia borchgrevinki *	Ross Sea	7M/3F
*Artedidraco skottsbergi *	Ross Sea	1M/6F

**Table 2:** Presence (**√**) or absence (×) of 5S rDNA (C1) and AFGP (C2) genes on the Y chromosome of Antarctic notothenioid species after FISH. In columns C3 and C4 diagrammatic representation of the chromosomes bearing the two probed genes. The chromosomal bands indicate the position of the 5S rDNA (C3) and AFGP (C4) genes resulting from FISH analysis. (—) data lacking.

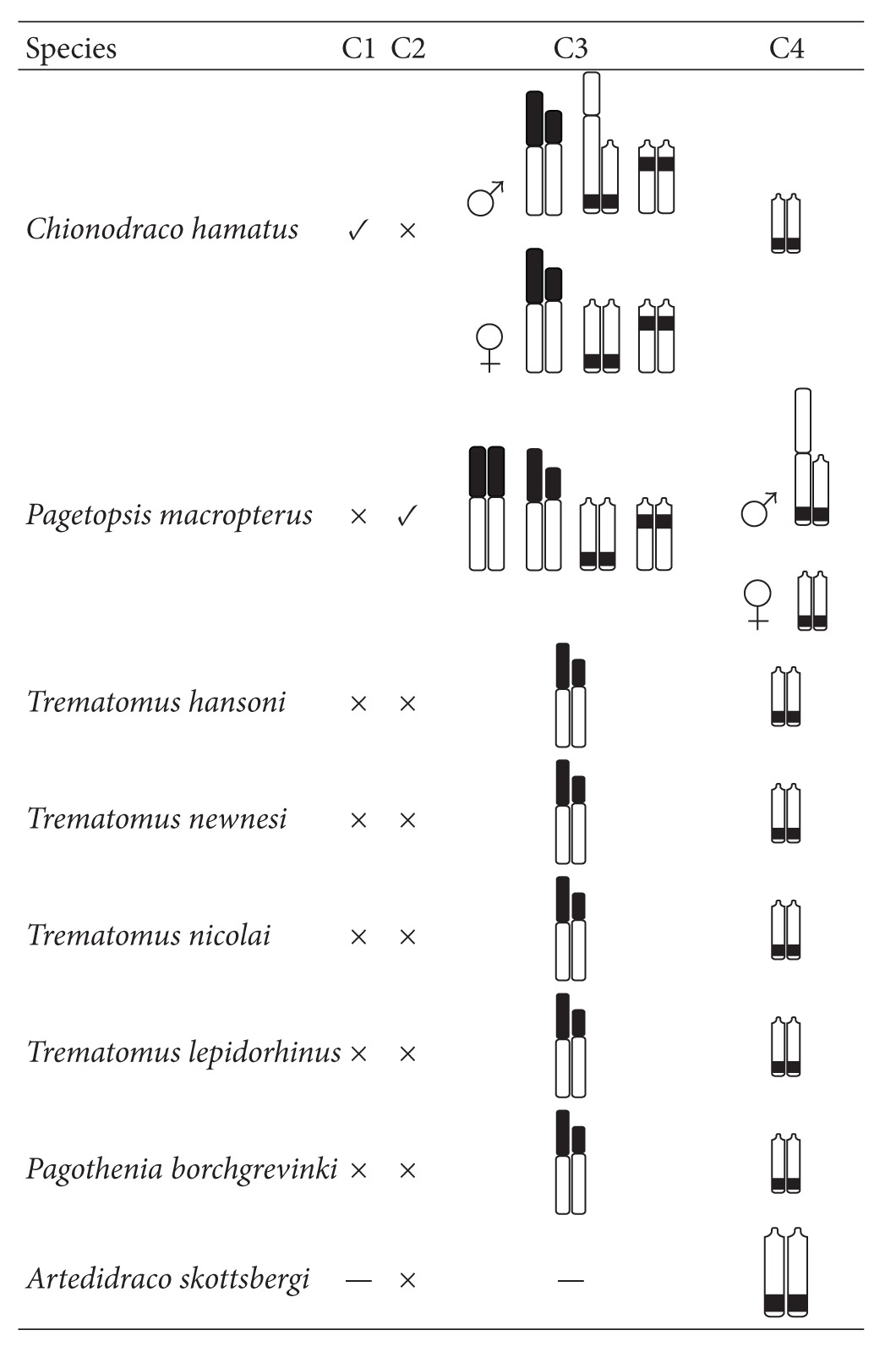

**Table 3:** Notothenioid fish species showing heteromorphic sex chromosomes.

Species	Reference
Nototheniidae	
* Pagothenia borgkrevinki *	[24]
* Trematomus hansoni *	[24]
* Trematomus lepidorhinus *	Pisano et al. (unpublished data)
* Trematomus newnesi *	[24]
* Trematomus nicolai *	[24]
Bathydraconidae	
* Bathydraco marri *	[25]
Artedidraconidae	
* Artedidraco skottsbergi *	[15]
Channicthyidae	
* Chaenodraco wilsoni *	[26]
* Chionodraco myersi *	[26]
* Chionodraco hamatus *	[27]
* Chionobathyscus dewitti *	[26]
* Pagetopsis macropterus *	[27]
